# A heterochromatin inducing protein differentially recognizes self versus foreign genomes

**DOI:** 10.1371/journal.ppat.1009447

**Published:** 2021-03-17

**Authors:** Eric M. Burton, Ibukun A. Akinyemi, Tiffany R. Frey, Huanzhou Xu, Xiaofan Li, Lai Jing Su, Jizu Zhi, Michael T. McIntosh, Sumita Bhaduri-McIntosh

**Affiliations:** 1 Dept. of Microbiology and Immunology, Stony Brook University, Stony Brook, New York, United States of America; 2 Child Health Research Institute, Dept. of Pediatrics, University of Florida, Gainesville, Florida, United States of America; 3 Division of Infectious Disease, Dept. of Pediatrics, University of Florida, Gainesville, Florida, United States of America; 4 Dept of Pathology, Stony Brook University, Stony Brook, New York, United States of America; 5 Child Health Research Institute, Depts. of Pediatrics and of Molecular Genetics and Microbiology, University of Florida, Gainesville, Florida, United States of America; 6 Division of Infectious Disease, Depts. of Pediatrics and of Molecular Genetics and Microbiology, University of Florida, Gainesville, Florida, United States of America; University of Wisconsin, UNITED STATES

## Abstract

Krüppel-associated box-domain zinc finger protein (*KRAB*-*ZFP*) transcriptional repressors recruit TRIM28/KAP1 to heterochromatinize the mammalian genome while also guarding the host by silencing invading foreign genomes. However, how a *KRAB*-*ZFP* recognizes target sequences in the natural context of its own or foreign genomes is unclear. Our studies on B-lymphocytes permanently harboring the cancer-causing Epstein-Barr virus (EBV) have shown that SZF1, a KRAB-ZFP, binds to several lytic/replicative phase genes to silence them, thereby promoting the latent/quiescent phase of the virus. As a result, unless SZF1 and its binding partners are displaced from target regions on the viral genome, EBV remains dormant, i.e. refractory to lytic phase-inducing triggers. As SZF1 also heterochromatinizes the cellular genome, we performed in situ footprint mapping on both viral and host genomes in physically separated B-lymphocytes bearing latent or replicative/active EBV genomes. By analyzing footprints, we learned that SZF1 recognizes the host genome through a repeat sequence-bearing motif near centromeres. Remarkably, SZF1 does not use this motif to recognize the EBV genome. Instead, it uses distinct binding sites that lack obvious similarities to each other or the above motif, to silence the viral genome. Virus mutagenesis studies show that these distinct binding sites are not only key to maintaining the established latent phase but also silencing the lytic phase in newly-infected cells, thus enabling the virus to establish latency and transform cells. Notably, these binding sites on the viral genome, when also present on the human genome, are not used by SZF1 to silence host genes during latency. This differential approach towards target site recognition may reflect a strategy by which the host silences and regulates genomes of persistent invaders without jeopardizing its own homeostasis.

## Author summary

Heterochromatin marks silenced portions of the human genome. Heterochromatin also serves as a defense strategy to silence foreign genomes. Yet, how the heterochromatin inducing KRAB-ZFP-TRIM28 machinery recognizes target sites on the native genome, whether self or foreign, is unclear. Using Epstein-Barr virus-infected cells in which a KRAB-ZFP, SZF1, silences lytic/replicative-phase genes of the virus, we performed in situ mapping of ZFP-footprints on cell and viral genomes. We find that while the ZFP uses a repeat sequence-bearing motif to target pericentromeric regions, it uses non-consensus sites to target viral genes. These findings point towards i) a mechanism for directing constitutive heterochromatin and ii) a strategy that allows the host to use the same heterochromatin machinery to regulate an invader without deregulating itself.

## Introduction

Heterochromatin mediates functions ranging from gene regulation and regulation of imprinting control regions and endogenous retroviruses to maintenance of stem cell pluripotency [1–4]. Heterochromatin also shields pericentromeric regions and telomeres [5]. To induce such heterochromatin, marked by the histone modification H3K9me3, a key scaffold protein called TRIM28 (tripartite motif protein 28)/KAP1 (KRAB-associated protein 1) recruits multiple histone-modifying proteins to DNA [6–8]. However, since TRIM28 is unable to bind DNA directly, targeting TRIM28 to DNA generally requires interaction with a KRAB-ZFP. KRAB-ZFPs (Krüppel-associated box-domain zinc finger proteins), members of the largest family of mammalian transcriptional repressors, consist of two functional components: zinc finger modules that bind to DNA and a KRAB domain that recruits TRIM28. Such TRIM28/KRAB-ZFP complexes mediate heterochromatin formation resulting in epigenetic silencing of gene transcription as well as protection of homologous sequences from recombination [8]. Recent findings show that in a defense strategy, TRIM28/KRAB-ZFP complexes also epigenetically silence foreign genomes and in the process, regulate the life cycle of certain viruses. These include extrachromosomal genomes of persistent viruses such as Epstein-Barr virus (EBV), Kaposi’s Sarcoma-Associated virus (KSHV), and human cytomegalovirus (CMV) [9–16]. Specifically for EBV and KSHV, we have previously shown that recruitment of TRIM28 to multiple viral lytic genes silences the destructive lytic program, thereby allowing these viruses to maintain latency. Disruption of TRIM28 binding to histone-modifying proteins, depletion of TRIM28, or depletion of SZF1 (Stem Cell Zinc Finger Protein 1, also known as ZNF589), the KRAB-ZFP that recruits TRIM28 to EBV and KSHV genomes, derepresses viral lytic genes by altering the heterochromatin status (H3K9Me3 and H3Ac), thus ending viral latency [10–13]. Thus, SZF1-TRIM28 complexes silence lytic genes on herpesviral genomes; this limits the pathology to the host while allowing the virus to persist in a latent/quiescent state.

Though TRIM28/KRAB-ZFP complexes engage both self and foreign genomes, how KRAB-ZFPs recognize target sequences on DNA in the natural context of the genome remains poorly understood. Further, whether KRAB-ZFPs like SZF1 target host and foreign genomes in a similar manner is also not known. While binding sites of some KRAB-ZFPs have been informatically inferred from ChIP-seq experiments utilizing overexpressed and tagged KRAB-ZFPs [17], defined through extracellular interactions with oligonucleotide libraries, or predicted through modeling [2,4,18–20], experimental identification of in situ footprints of KRAB-ZFPs on the genome is lacking. Using SZF1, we investigated how target sites on self and foreign genomes are recognized in EBV-infected B-lymphocytes. EBV infects greater than 95% of humans, persisting extra-chromosomally as multicopy episomes in B cells. Besides causing infectious mononucleosis, EBV can also cause B cell and epithelial cell cancers. While EBV remains quiescent in a latent state in B cells through expression of latency genes and partly through SZF1-TRIM28 mediated silencing of lytic genes, the virus can be reactivated into a productive/lytic phase by derepression of lytic genes. This productive phase is essential for EBV to persist in the population as well as to cause disease.

Using ChIP-exo, a high-resolution in situ strategy on isolated pure subpopulations of EBV-infected cells harboring latent versus lytic virus, we mapped SZF1 footprints on both self and viral genomes. Our analysis of the footprints on cellular genomes revealed a motif that was able to regulate gene expression. Footprints bearing this motif contained a repeat sequence and were enriched on pericentromeric regions of chromosomes. In contrast, viral genomes demonstrated no footprints bearing this motif. Experiments using virus mutants confirmed that SZF1 instead uses distinct non-consensus sequences to silence lytic genes on viral genomes, thereby shifting the balance towards latency. We found that such lytic silencing via interactions between SZF1 and non-consensus binding sites on the viral genomes is also needed to establish viral latency and transform newly infected B cells. Overall, the number of SZF1 footprints on the cell genome did not differ greatly between cells harboring latent or lytic viral genomes. That said, we found SZF1 to be enriched at the latent origin of replication (oriP) in lytic cells, though not contributing actively to viral genome replication arising from the lytic origins. The abovementioned non-consensus SZF1 binding sites identified on the viral genome were preferentially enriched in latent versus lytic cells, pointing towards SZF1-mediated differential host gene programming during latent and lytic states. Remarkably, although these non-consensus sites also mapped to the cell genome, they were not used by SZF1 to silence the host during latency. Thus, compared to self-genomes, the SZF1-TRIM28 machinery silences foreign herpesviral genomes using a different targeting strategy that may allow the host to shield itself from collateral damage.

## Results

### Mapping of SZF1 footprints and identification of candidate binding sites on the EBV genome

**Fig 1:**
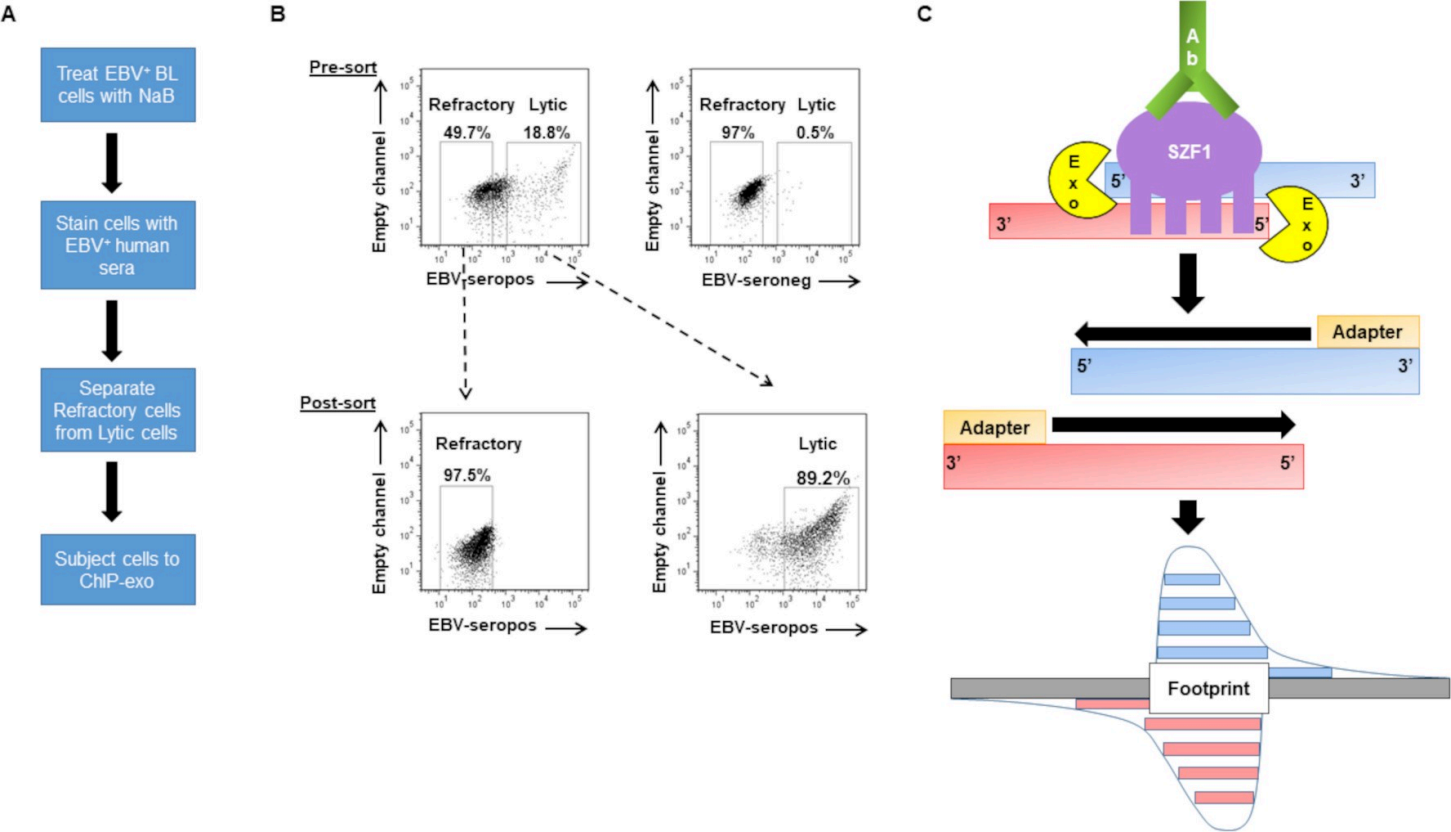
ChIP-exo in sorted-refractory cells to map genome-wide SZF1-binding sites. (**A**) Experimental design for isolation of EBV-positive refractory and lytic cells for ChIP (chromatin immunoprecipitation)-exo protocol. (**B**) Pre-sort and post-sort analysis of FACS separation of refractory and lytic cells. EBV-positive HH514-16 BL cells were treated with NaB for 24 hours and harvested for flow sorting. (B, top) Reference EBV-seropositive serum was used to demarcate lytic cells and EBV-seronegative serum was used as negative control for gating purposes. (B, bottom) Post-sort analysis was performed to confirm purity and efficacy of sort. (**C**) Illustration of ChIP-exo protocol for SZF1 in sorted cells. DNA immunoprecipitated by anti-SZF1 antibody is treated with a 5’-to-3’ exonuclease (Exo) while still in the immunoprecipitate. The 5’ ends of digested DNA are concentrated at a fixed distance from the sites of crosslinking (i.e. footprint) and are detected by deep sequencing.

To identify SZF1’s footprints in the genomic context, we combined two approaches. The first is a cell-separation strategy that we developed to isolate latent cells in which EBV is refractory to lytic triggers from those that support the EBV lytic phase [21,22]–this was important because compared to lytic cells, refractory cells demonstrate preferential silencing of viral lytic genes by SZF1 [12,22]. The second is ChIP-exo, a ChIP-seq technique paired with exonuclease excision, which precisely maps and sequences fragments of the genome to which the protein of interest is bound [23]. Of several EBV^+^ tumor/transformed B cell lines that were available, we chose HH514-16, a well-studied Burkitt lymphoma (BL)-derived cell line because these cells are tightly latent at baseline but can be readily triggered to support the lytic phase. As shown in Fig 1A and 1B, after exposing these cells to a lytic trigger, our cell-separation strategy allowed the separation of cells in which EBV was refractory from those poised to support the lytic phase. We carried out ChIP-exo in sorted cells using an SZF1-specific antibody and subjected precipitated chromatin to deep sequencing. Using a previously described bioinformatic analysis pipeline [24], we then identified DNA sequence reads that contributed to footprint regions obscured by SZF1 (Fig 1C). S1 Table shows ChIP-exo reads that mapped to human and EBV genomes. S2 Table shows mapping of indexed reads to ChIP-exo peaks, i.e. SZF1 footprints, on human and EBV genomes in cells unexposed to the lytic trigger (untreated), exposed to the lytic trigger but remained latent (refractory), or switched into the lytic phase in response to the lytic trigger (lytic). On the whole, the three subpopulations of cells showed only 2 to 5-fold differences in footprint/peak numbers across the human genome, which, given the size of the human genome, is not large. With regard to the viral genome, cells with lytic virus had more reads and footprints compared to refractory and untreated cells, possibly due to an increase in lytic virus replication.

**Fig 2:**
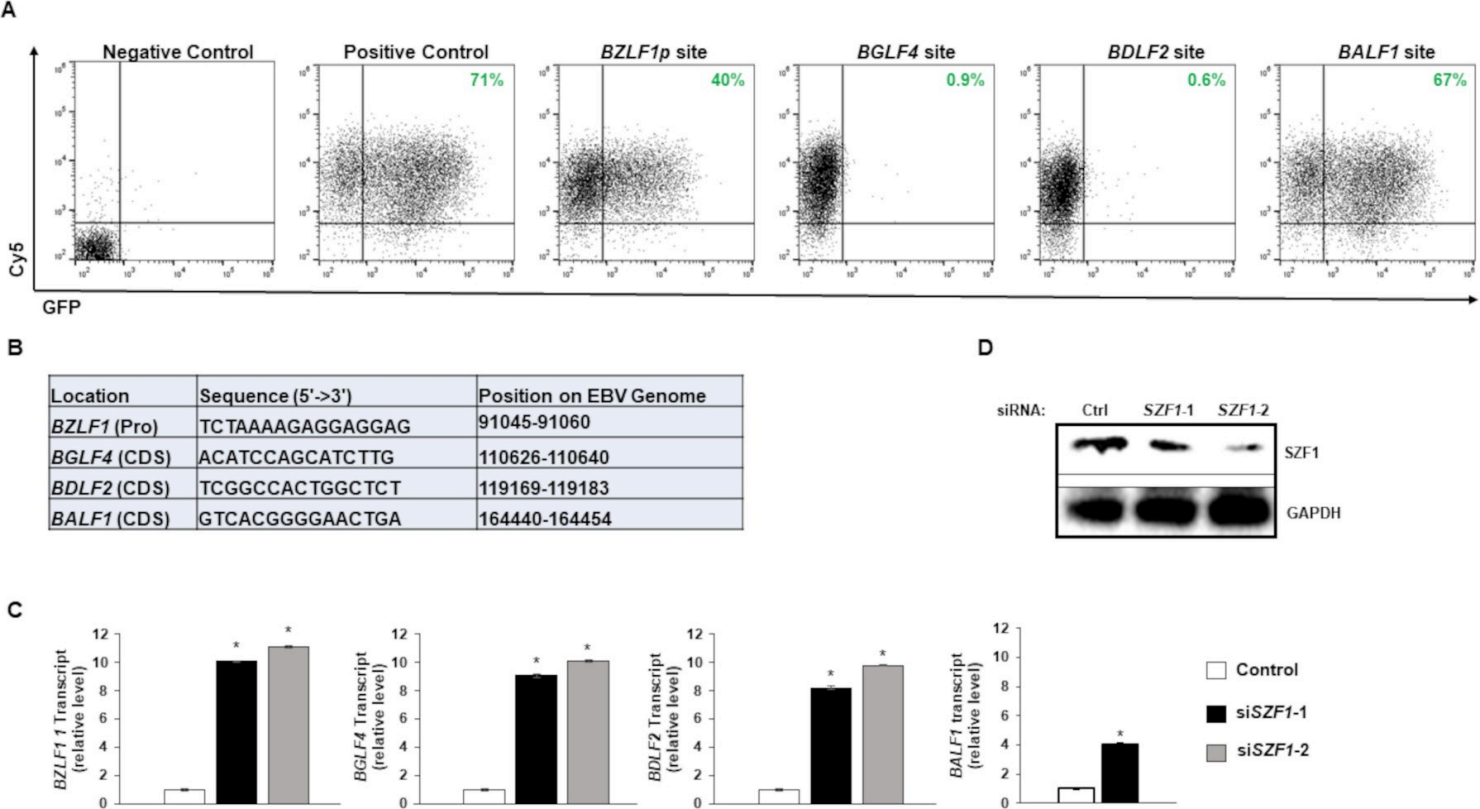
Effects of EBV genome-derived candidate SZF1-binding sites on extrachromosomal gene expression. (**A**) Candidate SZF1-binding site fragments from the EBV genome were cloned into pEGFP-N1 vector. SZF1-binding site pEGFP-N1 vectors or control pEGPFn1 vector (positive control) were transfected into HEK293T cells and assayed for relative GFP expression by flow cytometry. In addition, Cy5-non-targeting siRNA was co-transfected to monitor transfection efficiency between samples. Empty vector and non-fluorescent, non-targeting control siRNA-transfected 293T cells were used as negative control and for gating purposes. Percent cells expressing GFP are labeled in green. (**B**) Sequences of candidate SZF1-binding sites identified via ChIP-exo and tested in panel A. Positions on the EBV genome and with respect to nearby “target” genes are also shown; Pro, promoter; CDS, coding sequence. (**C**) Knockdown of *SZF1* using two separate siRNAs or a control non-targeting siRNA was performed in HH514-16 BL cells. After 24 hours, NaB was added to activate EBV lytic cycle. After another 24 hours, cells were harvested for RNA extraction and RT-qPCR analysis was performed for relative EBV lytic transcripts in control non-targeting siRNA-transfected cells (white bar) versus two distinct siRNAs targeting *SZF1* (black and grey bars). Data represent averages of three independent experiments; error bars, SEM; *, p ≤ 0.05. (**D**) Twenty-four hours after transfection, cells from (C) were harvested for immunoblot to validate SZF1 knockdown.

With our earlier work showing that SZF1 enforces the refractory/latent state by silencing EBV lytic genes [12], we focused on footprints of SZF1 on the EBV genome in refractory cells. We identified 31nt SZF1 footprints that were within lytic genes (and extending 500bp upstream) and were enriched in the SZF1 pull-down from refractory cells (S3 Table). These 31nt footprints represented merged overlapping peaks from both DNA strands and included putative SZF1-binding sites; SZF1 has 4 zinc fingers, each able to bind 3 nucleotides, resulting in a predicted binding site of 12nt. Informatic analysis of these footprints on the viral genome did not reveal any obvious consensus sequences or motifs. We therefore selected eight footprints with high read counts that mapped to lytic genes of each kinetic (immediate early, early, and late) class of the EBV lytic phase (S3 Table); each footprint was fragmented into 3 overlapping pieces (15-16nt each) that were tested individually for their repressive ability when placed upstream of a GFP cassette. Previous evidence suggests that KRAB-ZFPs can bind to and regulate plasmid DNA [16]. Fig 2A and 2B show three fragments corresponding to the *BZLF1* promoter (*BZLF1p*, immediate early lytic gene), *BGLF4* (early lytic) gene, and *BDLF2* (late lytic) gene, respectively, that demonstrated between 44% and 99% repression of GFP. In contrast, the others did not silence GFP; an example of one such site associated with *BALF1* is also shown (Fig 2A). Corroborating the findings in Fig 2A, knockdown of *SZF1* derepressed target gene expression, i.e. from *BZLF1*, *BGLF4*, and *BDLF2* (Fig 2C and 2D)–though likely through a combination of direct and indirect effects in the cases of *BGLF4* and *BDLF2*, as the *BZLF1* gene product (ZEBRA) can transcriptionally activate kinetically downstream lytic genes [25]. However, despite this anticipated indirect effect of ZEBRA on the early gene product *BALF1*, we observed a blunted *BALF1* transcriptional response to si*SZF1* compared to those of *BGLF4* and *BDLF2* (Fig 2C), agreeing with the inability of the *BALF1*-related candidate sequence to silence GFP expression. Thus, several candidate SZF1-binding sites were capable of silencing extrachromosomal gene expression.

### SZF1 uses distinct binding sites to silence EBV genes that disrupt the latent state

**Fig 3:**
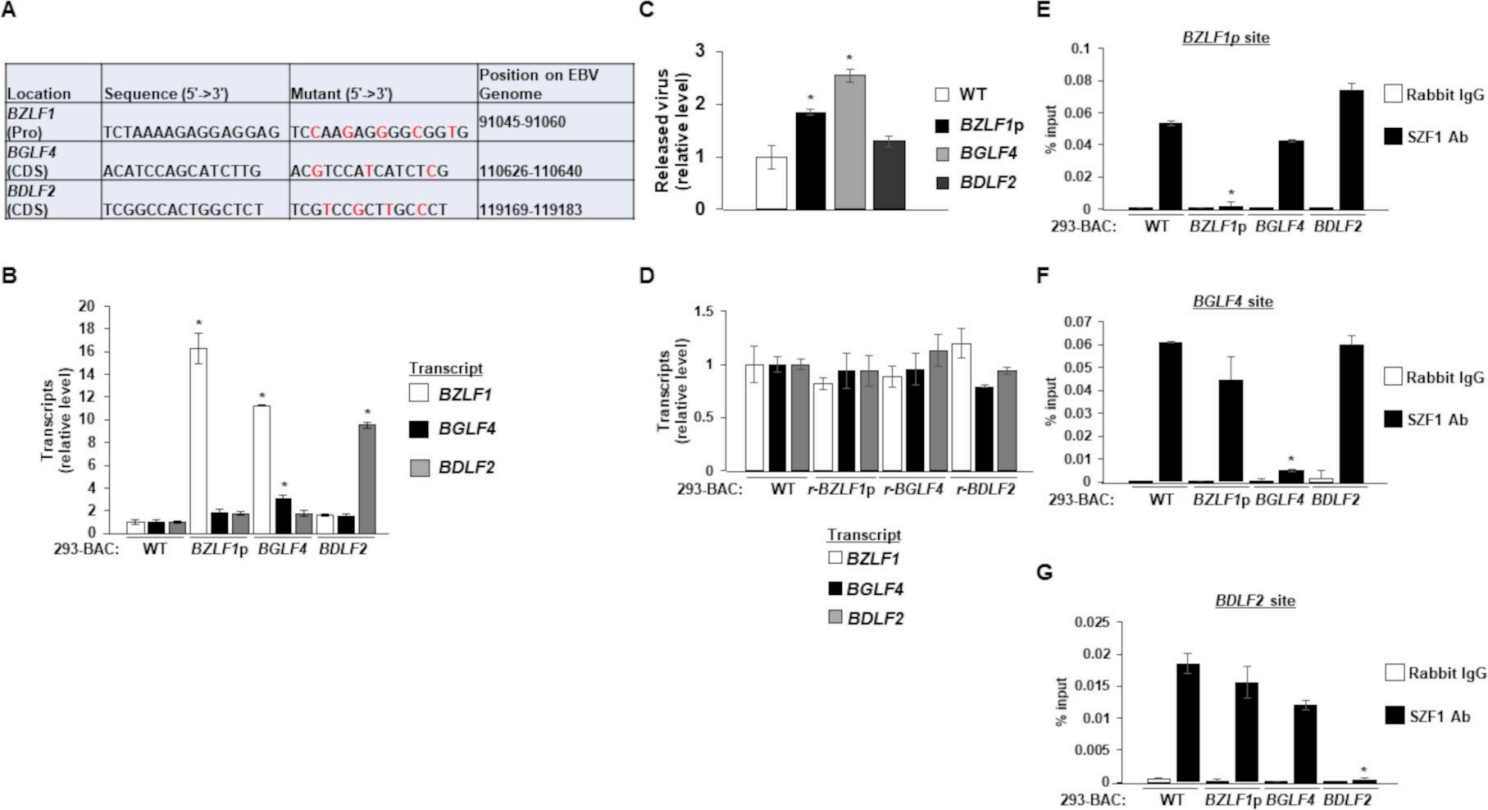
Mutations in candidate SZF1-binding sites derepress EBV lytic genes. (**A**) Synonymous point mutations were made in candidate SZF1-binding sites on the p2089 BACmid via red recombineering. Mutant residues are shown in red. (**B**) After transfection and hygromycin selection (~ 2 weeks later), 293-BAC cells harboring wild-type p2089 BACmid or BACmids with mutant SZF1-binding sites were harvested for RNA extraction and RT-qPCR analysis for relative expression of EBV lytic genes (from each kinetic class: *BZLF1*, immediate early; *BGLF4*, early; *BDLF2*, late), compared to wild-type BAC sample. (**C**) Supernatants from 293-BAC cells harboring p2089-BACs bearing *BZLF1* promoter, *BGLF4* coding sequence, or *BDLF2* coding sequence SZF1-binding site mutations were harvested and analyzed via qPCR for relative amounts of released DNase-resistant virus compared to the wild-type 293-BAC sample. (**D**) 293-BAC cells harboring wild-type p2089-BAC or p2089 BACmids that underwent reversion (r) mutations for their respective SZF1-binding sites were tested by RT-qPCR of lytic genes *BZLF1*, *BGLF4*, and *BDLF2* relative to wild-type 293-BAC. (**E-G**) SZF1-ChIP was performed on wild-type 293-BAC samples or 293-BACs harboring SZF1-binding site mutations; precipitated chromatin was analyzed via qPCR using primers to amplify PCR products flanking the putative *BZLF1* promoter sequence (E), the *BGLF4* coding sequence site (F), or the *BDLF2* coding sequence site (G). ChIP-PCR results were analyzed relative to 1% input and displayed as percent input. Data represent averages of three independent experiments; error bars, SEM; *, p ≤ 0.05.

With repressive effects confirmed at a GFP locus, we investigated the ability of the three candidate SZF1-binding sites to silence their respective loci on the EBV genome; notably, the three binding sites appeared distinct from each other. To avoid changes to amino acid composition of products encoded by the target genes *BGLF4* and *BDLF2*, we used red recombineering to make synonymous point mutations to their respective candidate SZF1-binding sites on the p2089 EBV BACmid (Fig 3A) [26,27]; the binding sites on *BGLF4* and *BDLF2* did not overlap with other known ORFs. In the case of *BZLF1*, the binding site was within its promoter but in an intron of the overlapping gene *BRLF1*. To generate virus-producing cell lines, we introduced the wild-type p2089 BACmid or mutated p2089 BACmids into HEK-293T cells and selected with hygromycin to establish 293T cells harboring the EBV episome, henceforth known as 293-BAC cells. Using RT-qPCR to assay the effects of mutations in candidate SZF1-binding sites, we saw significant increases in the respective target genes when binding sites were mutated compared to wild-type EBV (Fig 3B). We also witnessed slight increases in the expression of other lytic genes in this *BZLF1p* mutant, although the results were not statistically significant. Of note, we detected not only increased *BGLF4* transcripts in the *BGLF4* SZF1-binding site mutant but also elevated *BZLF1* transcripts; this is consistent with previous results from our lab showing that vPK, the product of *BGLF4* can upregulate *BZLF1* in a retrograde manner (Fig 3B) [13]. As expected however, derepressing the late gene *BDLF2* had no effect on *BZLF1* and *BGLF4* expression.

To assess if derepression of EBV genes caused by mutations in SZF1-binding sites was sufficient to disrupt latency, we assayed virus release and detected modest but significant increases in released EBV in the supernatants from the *BZLF1p* and *BGLF4* SZF1-binding site mutants compared to WT 293-p2089 cells (Fig 3C). Not surprisingly, derepression of a late lytic gene (*BDLF2*) was not sufficient to disrupt latency in the absence of expression of viral genes from kinetically earlier stages of the lytic phase (Fig 3C). To minimize the possibility that off-target changes during red recombineering were responsible for derepressing EBV genes, we made reversion mutations to the three mutated p2089 BACmids and introduced them back into HEK-293T cells. Confirming that mutations in SZF1-binding sites were indeed responsible for observed outcomes in Fig 3B and 3C, revertant mutants were unable to derepress target genes (Fig 3D). To test if SZF1 localized to candidate SZF1-binding sites, we used an SZF1-antibody for chromatin immunoprecipitation (ChIP)-PCR and as expected, observed significantly less enrichment of SZF1 on the target gene corresponding to the mutant (compared to wild-type virus) but not on the targets for the other sites in question (Fig 3E-G). Taken together, these results indicate that the SZF1 repressor localizes to candidate SZF1-binding sites to silence target lytic genes on the EBV genome, and, loss of this enrichment disrupts the EBV latent state. Moreover, interactions of SZF1 to DNA are site-specific and loss of SZF1 binding due to modification of a particular binding site is specific for the site in question without affecting binding to other sites.

### Viruses with mutant genomes unable to interact with SZF1 are spontaneously lytic and defective in transforming B-lymphocytes

**Fig 4:**
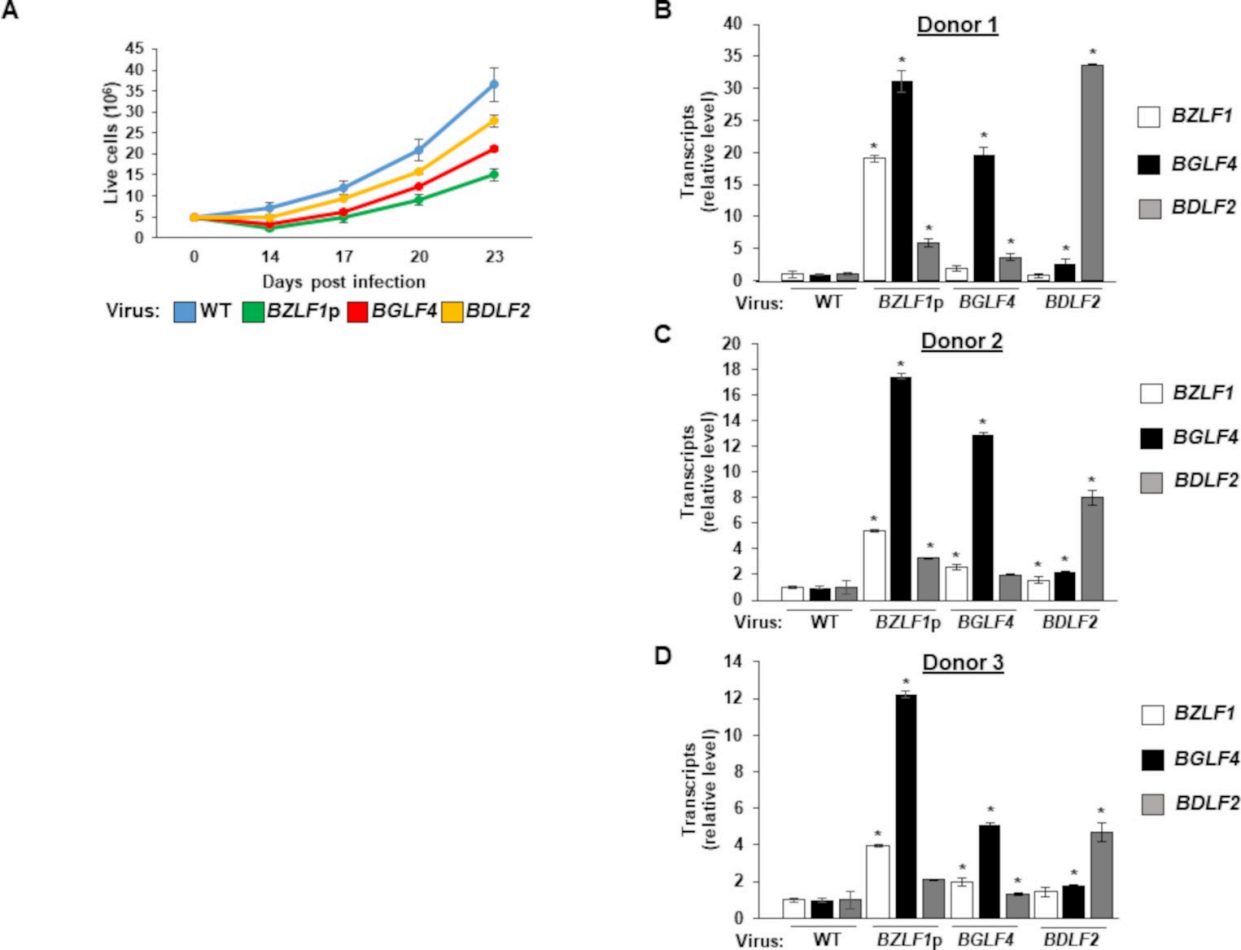
Viruses harboring mutated SZF1-binding sites spontaneously express lytic genes and demonstrate defects in B cell transformation. (**A**) Peripheral blood mononuclear cells (PBMC) from three healthy donors were infected with wild-type or SZF1-binding site mutant p2089 viruses in the presence of FK506. Cells were counted using Trypan Blue at indicated time points and absolute live cells were plotted. Results were averaged between three donors. (**B-D**) PBMC from 3 donors infected as in (A) were harvested after 48 hours for RT-qPCR analysis of EBV lytic genes *BZLF1*, *BGFL4*, and *BDLF2*, relative to wild-type p2089-infected PBMC. Data represent averages from three independent experiments; error bars, SEM; *, p ≤ 0.05.

Silencing the lytic program is an important step in establishing EBV latency and B cell transformation. With SZF1-binding site mutant EBV unable to silence target lytic genes, we examined the ability of such mutants to transform primary B-lymphocytes. We infected primary B-lymphocytes from three healthy subjects with wild type or mutant EBV at MOI of 1 in the presence of FK506 (to inhibit T cells) and assayed for outgrowth of culture, i.e. transformation. We followed the kinetics of LCL outgrowth and observed substantial delays compared to the wild-type virus, with cells infected with *BZLF1*p site mutant virus slowest to grow followed by *BGLF4* and *BDLF2* site mutant viruses (Fig 4A).

We investigated if delayed transformation by viruses bearing SZF1-binding site mutants was related to defective silencing of lytic genes. Similar to results obtained in 293-BAC cells (Fig 3B and 3C), as early as 48 hours after infection, we observed increased expression from target genes corresponding to mutated SZF1-binding sites. Notably, we saw substantial increases in all 3 EBV lytic genes representative of immediate early, early, and late kinetic classes from the *BZLF1*p site mutant in all three donors and modest increases in immediate early and late gene expression in the *BGLF4* site mutant virus-infected samples (Fig 4B–4D). The *BDLF2* site mutant virus mainly increased the amounts of *BDLF2* transcripts in comparison to the wild type p2089 virus, although there were modest increases in *BZLF1* and *BGLF4* transcripts in some of the samples (Fig 4B–4D). These results indicate that candidate SZF1-binding sites are important not only for silencing viral genes essential for the lytic program but also to ensure transformation, and thereby establishment of latency in B cells.

### Validated SZF1-binding sites from the viral genome are predicted or footprinted on the human genome but are not used by SZF1 to silence host genes during latency

**Fig 5:**
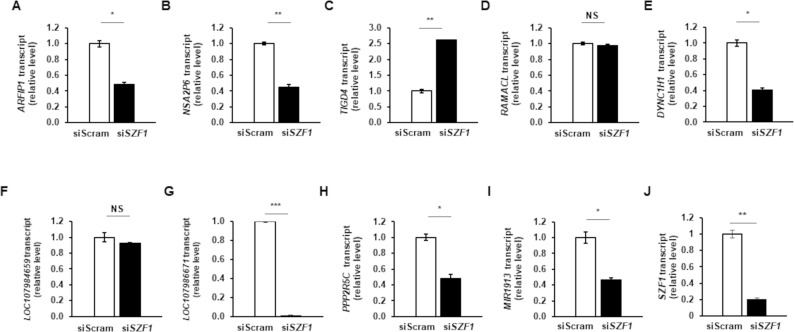
Binding sites used by SZF1 to silence EBV lytic genes are not used to repress host genes during latency. SZF1 was depleted using a validated siRNA or a control non-targeting siRNA (siScram) in HH514-16 BL cells. After 24 hours, NaB was added to activate EBV lytic cycle. After another 24 hours, cells were harvested for RNA extraction and RT-qPCR analysis of host genes harboring the *BZLF1*p binding site (**A-C**) or the *BDLF2* binding site (**D-I**). Data represent averages of two independent experiments; error bars, SEM; *, p < 0.05; **, p < 0.01; ***, p < 0.001.

With SZF1 binding and function confirmed at three distinct sites, we asked if these sites also existed and were bound by SZF1 on the cellular genome. We found that contiguous stretches of 12nt from *BZLF1*p, *BGLF4*, and *BDLF2* binding sites matched to and footprinted at three, one, and three genes, respectively, in the human reference genome Hg38 (S4 Table). Of these seven, five genes, all on different chromosomes but bearing the *BZLF1*p or *BDLF2* binding site were footprinted by SZF1 preferentially in refractory cells. To test if SZF1 uses these non-consensus distinct binding sites to also silence the cellular genome during latency, we depleted SZF1 using an siRNA (validated in Fig 2) in lytically induced cells. Of the five genes, we observed expression of only two following lytic activation; however, contrary to expectation, expression of both *ARFIP1* and *PPP2R5C* was decreased during the lytic phase when SZF1 was depleted (Fig 5A and 5H). Of the remaining three, expression from *ENOX1* and *RPS6KA2* was not detected during the lytic phase while *CACNA1E* expression was not detectable in uninduced or induced cells. We also tested expression of seven other genes/loci located within 100kb of SZF1-footprinted sites preferentially identified in refractory cells, and found that all except one demonstrated no change or repression when SZF1 was depleted in cells exposed to lytic trigger (Fig 5B, 5D, 5E, 5F, 5G, 5I, and 5J). The exception, *TIGD4*, demonstrated a 2.5-fold increase upon SZF1 depletion (Fig 5C), a mild effect compared to those observed on viral gene targets *BZLF1*, *BGLF4*, and *BDLF2*, shown in Fig 2C. Thus, SZF1 does not appear to use the non-consensus distinct binding sites, identified from the viral genome, to silence host genes during latency.

### SZF1 footprints on the B cell genome reveal a consensus distinct from validated sites on the viral genome

**Fig 6:**
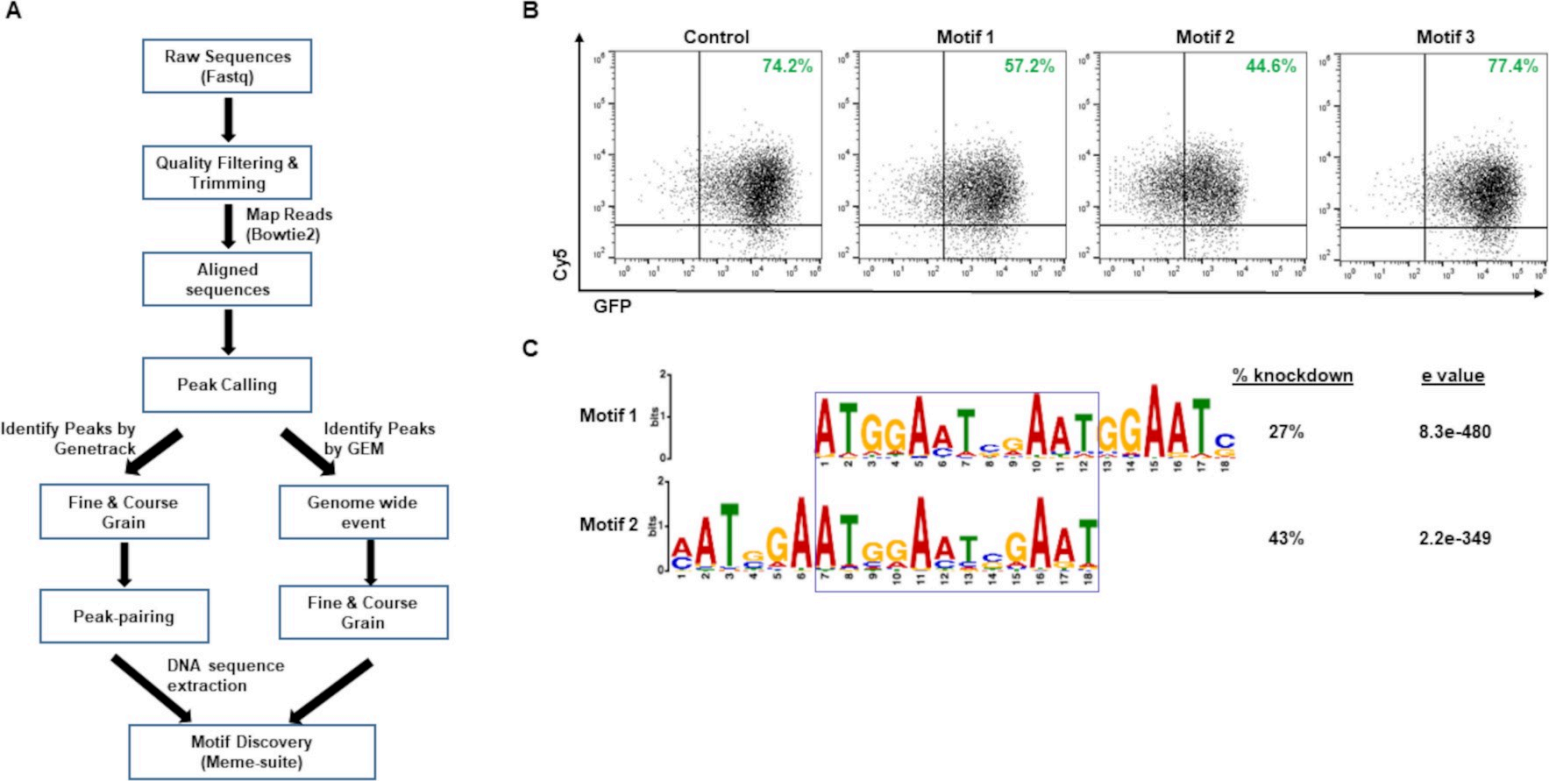
Evaluation of SZF1-footprint derived motifs on the B cell genome. (**A**) Informatic workflow for SZF1 motif discovery. (**B**) Informatically-derived putative SZF1-binding motif consensus sequences were cloned into a pEGFP-N1 vector and transfected into HEK293T cells. GFP expression was assessed relative to control pEGFP-N1 vector lacking putative SZF1 motif consensus sequences; Cy5-non-targeting siRNA was co-transfected to monitor transfection efficiency. Percent GFP^+^ cells from three of 27 motif-consensus-sequence-bearing cassettes are displayed in green; only consensus sequences derived from motifs 1 and 2 repressed GFP expression, and motif 3 is a consensus sequence representative of the remaining 24 motifs. (**C**) SZF1 motifs derived from Meme-suite. Motif logos of the two motifs capable of repressing GFP in (B) were aligned and overlap demarcated by a box. Percent GFP knockdown in (B) and statistical significance of motif determined using Meme-suite are shown.

While we found specific regions on the EBV genome that are required for SZF1 to silence the lytic program, we were unable to find a consensus by comparing the three validated binding sites and their reverse complements. However, with the abundance of reads from the human genome, we were able to identify SZF1-binding motifs across primarily the B cell genome using the strategy outlined in Fig 6A. We found several motifs that were abundantly represented with high statistical significance (S5 Table). To validate, we cloned the 27 most statistically significant consensus sequences into a pEGFP-N1 vector as before (Fig 2) and assayed their ability to silence GFP. As shown in Fig 6B, only consensus sequences for motifs 1 and 2 blunted GFP expression in comparison to the wild type GFP plasmid; motif 3 is an example of motifs that did not alter GFP expression. Consensus sequences from motifs 1 and 2 demonstrated 27% and 43% knockdown respectively in percent GFP^+^ cells and reduced the intensity of GFP staining of the predominant population of cells. Upon closer inspection of motifs 1 and 2, we found a 12nt overlap/consensus that contained a 5-8nt repeat region of AAT G/C G/A AAT (Fig 6C). Although four other motifs in S5 Table contained the sequence AATGGAAT, none of the consensus sequences derived from these motifs silenced GFP expression. By comparison, motifs 1 and 2, containing both AATGGAAT and AATCGAAT, whether overlapping or near each other, were able to silence GFP. While AATGGAAT, more commonly observed among the motifs in S5 Table, represented 1% of the mapped reads, AATCGAAT constituted only 0.1% of reads pulled down by SZF1, regardless of whether cells were untreated, refractory, or lytic (S6 Table). Remarkably, none of the three validated SZF1-binding sites from the EBV genome were similar to motifs identified from the B cell genome-derived sequences. These results indicate that SZF1 uses at least one consensus sequence to target the human genome.

**Fig 7:**
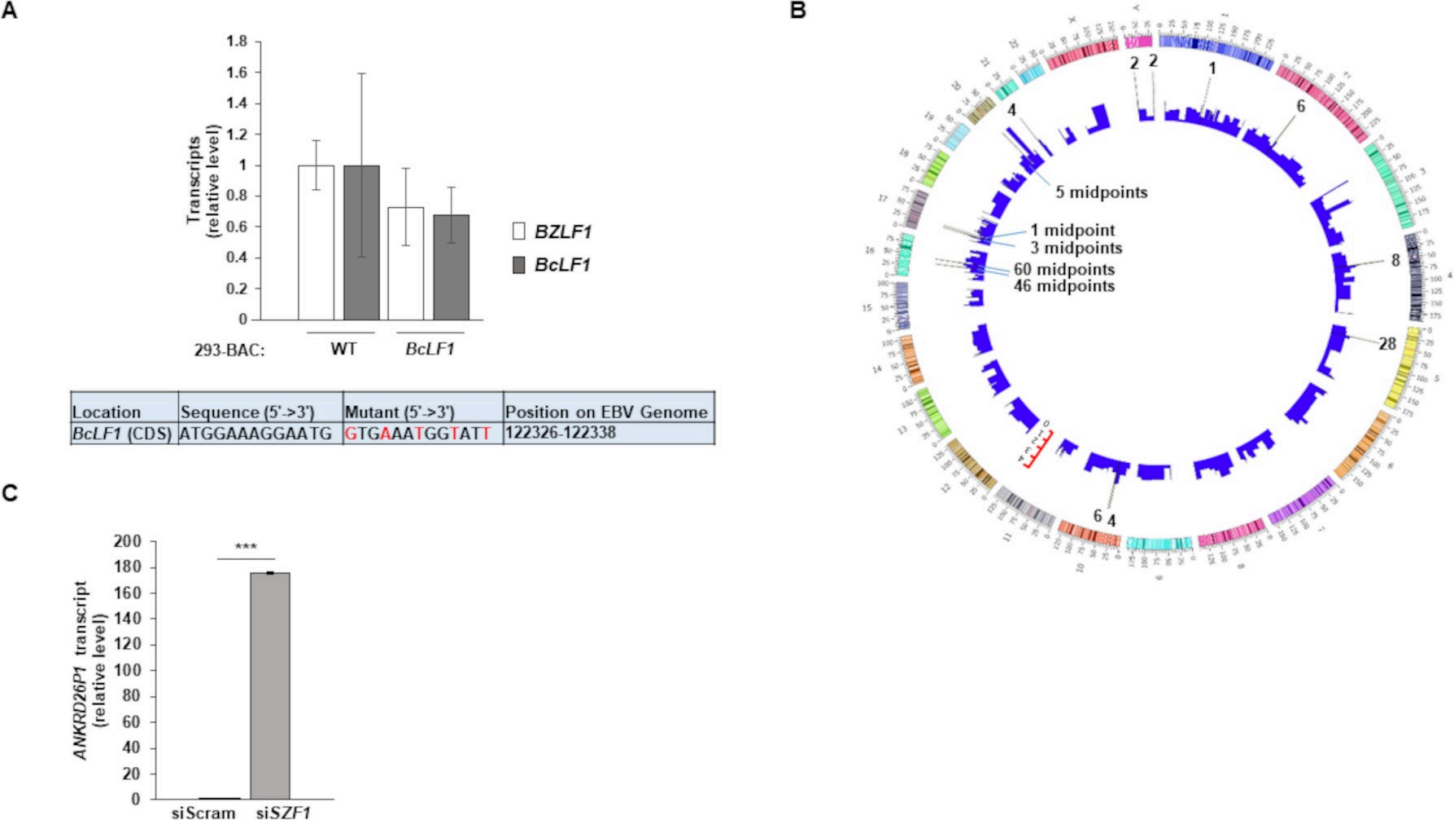
SZF1 binding motif 1 on viral versus host genomes. (**A**) A sequence matching the consensus of motif 1 identified within the *BcLF1* gene of the EBV genome was mutated using red recombineering. This mutant and wild-type p2089 BAC were transfected into HEK293T cells. Cells were harvested for RT-qPCR analysis of EBV lytic genes *BZLF1* and *BcLF1*. Data represent averages of three independent experiments; error bars, SEM. (**B**) Circos plot showing SZF1-footprints across the human genome and the locations of SZF1 binding motif 1. Height of bars represent indexed read counts; read counts are presented in Log10, sigma (s) = 5, exclusion zone = 10 and allowing no singleton. The spikes represent 176 peak-pair midpoints that contributed to the generation of SZF1 binding motif 1. Scales next to chromosomes indicates length of chromosomes. (**C**) Validated targeting si*SZF1*-2 from Fig 2 or a control non-targeting siRNA (siScram) was introduced into HH514-16 BL cells. After 24 hours, cells were harvested and RT-qPCR analysis performed for relative levels of *ANKRD26P1* transcript. Data represent averages of two independent experiments with three technical repeats each; error bars, SEM; **, p < 0.01; ***, p < 0.001.

When we searched the EBV genome, we only found one site that matched 13nt and 12nt, respectively, of motifs 1 and 2 within the coding sequence of the *BcLF1* lytic gene. Although we did not identify this site as a SZF1-footprint in our ChIP-exo dataset, we nonetheless generated a *BcLF1* site virus mutant to assess if this consensus site contributed to *BcLF1* expression or expression of the latent-to-lytic switch gene *BZLF1*. We did not detect an increase in *BcLF1* or *BZLF1* expression (Fig 7A), indicating that the consensus site was not used for SZF1 binding on the viral genome.

Analysis of the host genome revealed that 176 SZF1 footprints bore the SZF1 consensus sequence, i.e. motif 1 (Fig 7B and S7 Table); we focused on the consensus sequence for motif 1 as it showed greater statistical significance and was inclusive of more peak midpoints than motif 2. We found that most of the footprints were pericentromeric with 60 and 46 footprints immediately adjacent to the centromere on the q and p arm, respectively of chromosome 16. Similarly, 28 footprints on chromosome 5 were also pericentromeric (Fig 7B). None of these footprints was within annotated genes or open reading frames. Since repressive effects of KRAB-ZFP-TRIM28 complexes are thought to extend several tens of kilobases [14], we searched for genes within 100kb on both sides of motif 1-bearing footprints on chromosomes 16 and 5 and found only one (S8 Table). This gene, *ANKRD26P1* is a pseudogene that expresses a protein of unknown function, primarily in plasma [28]. When we depleted SZF1 from cells (as shown in Fig 5J), we found significant derepression of *ANKRD26P1* (Fig 7C), suggesting that *ANKRD26P1* is silenced by SZF1. Whether this silencing is mediated in cis by SZF1 bound to motif 1 ~100kb away or in trans by distant regions of SZF1-bound chromatin looping close to the *ANKRD26P1* locus is unclear. Thus, SZF1 recognizes target regions on the cellular genome using a repeat sequence-bearing motif that is enriched at/near centromeres. Moreover, this motif is poorly represented and ineffective in silencing gene expression from the viral genome.

### SZF1 is enriched at oriP on lytic genomes of EBV

**Fig 8:**
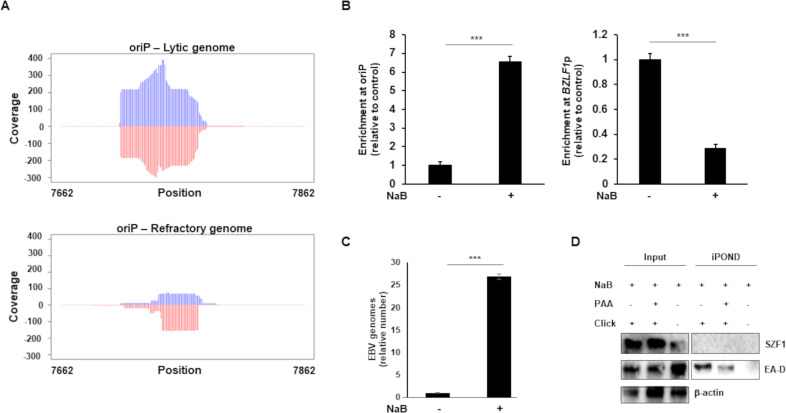
SZF1 is enriched at oriP on the lytic genome but is not associated with actively replicating viral DNA. **A.** Read coverage at EBV oriP. Plots show the read distributions at oriP under lytic and refractory conditions. Coverage of the reads from lytic and refractory genomes were determined with Bedtools software (v2.30.0) and plotted in R. Lytic reads were normalized to refractory EBV genome copy number, Rightward and leftward strands are indicated by blue and red, respectively. Genome position numbers corresponding to the reference genome NC_007605 are indicated. **B-D.** EBV-positive cells were untreated or exposed to NaB for 36 hours (or 24 hours in C) and subjected to (B) ChIP with anti-SZF1 antibody or control antibody followed by qPCR amplification of oriP and *BZLF1*p and analysis of data by normalizing to 2% input and IgG control, (C) isolation of intracellular DNA and qPCR amplification of *BALF5* gene, or (D) isolation of proteins on nascent DNA (iPOND) followed by western blotting with indicated antibodies. Data in B and C represent averages of two independent experiments; error bars, SEM; ***, p < 0.001; iPOND was performed twice.

As shown in S1 Table, a substantially greater number of SZF1 ChIP-exo reads mapped to the EBV genome from sorted lytic cells than from refractory or untreated cells. Not surprisingly, this appeared largely due to the presence of many more viral genomes in EBV lytic cells. Using the conservative estimate of 125 lytic genomes to 1 refractory genome based on a 25-fold increase in EBV copy number in induced versus uninduced cells (Fig 8C) and that only ~20% of unsorted NaB-induced cells harbored lytic replicating virus (Fig 1B), we normalized lytic read counts to the refractory genome copy number. This normalization revealed far fewer reads mapping per genome for lytic than refractory (S1 Table; normalized EBV mapped reads). Despite this reduction in read counts on a per genome basis, we noticed a cluster of normalized reads mapping to lytic EBV genomes uniquely located at the EBV oriP locus (Fig 8A). In contrast, normalized read distributions at the SZF1-binding sites for *BZLF1p*, *BGLF4*, and *BDLF2* showed far greater read counts on a per genome basis in refractory cells as compared to lytic (S1 Fig). Using ChIP-PCR, we were able to confirm significant preferential enrichment of SZF1 at oriP after lytic activation (Fig 8B). With SZF1 being a DNA binding protein and its association, albeit low level, observed throughout the viral genome in sorted lytic cells (S1 Fig), we also asked if SZF1 might contribute to viral DNA replication originating from the lytic origins of replication. We therefore isolated nascent DNA using iPOND [29] from lytic-trigger exposed cells and found that while EA-D, as expected, was enriched at viral replication forks, SZF1 was not; consistent with pull-down of nascent viral DNA, the presence of PAA, a viral DNA polymerase inhibitor, demonstrated a reduction in DNA-bound EA-D (Fig 8D). Thus, on lytic genomes, SZF1 i) is enriched at the latent origin of replication, known to be silent during the lytic phase and ii) is not associated with actively replicating viral DNA.

Overall, SZF1, a KRAB-ZFP repressor, can recognize the cellular genome via a repeat sequence-bearing motif. While SZF1 uses the motif to concentrate at pericentromeres, known to bear constitutive heterochromatin, and likely silences genes in the vicinity, it uses several distinct non-consensus sequences to silence lytic genes on the viral genome. Remarkably, SZF1 does not appear to silence host genes in EBV latency using these non-consensus sequences. This distinction between self and non-self allows the cell to silence/regulate EBV while simultaneously guarding its own genome.

## Discussion

This study describes how the machinery that induces chromosomal heterochromatin but also silences extrachromosomal foreign genomes is able to differentiate between self and non-self by targeting dissimilar binding sites. Through the use of a precise in situ strategy to map footprints of SZF1 on DNA, it provides an original view of some of the natural target sites of a KRAB-ZFP on both self and foreign genomes. It also identifies a motif using which SZF1 targets known regions of pericentromeric heterochromatin. While the bulk of constitutive heterochromatin surrounds centromeres, thus far no consensus sequence that causes nucleation of a KRAB-ZFP at pericentromeric regions has been described. We suspect that additional consensus binding sites exist on chromosomal DNA to help the heterochromatin machinery distinguish self from invaders; else, dysregulation of heterochromatin while regulating foreign genomes could provoke genetic turmoil by inducing aberrant repair of repeat sequences, chromosome missegregation, and transposon activation, all linked to aging and cancer development. Lastly, our findings indicate that the KRAB-ZFP-TRIM28 machinery not only keeps a common persistent virus in a subdued state within the cell, but it also silences viral genomes rapidly upon their entry into new cells.

From the virus’s standpoint, KRAB-ZFP-TRIM28-mediated silencing allows it to go into hiding upon entry into a new cell. Indeed, we find that virus mutants unable to bind SZF1 are more prone to disrupting latency but also less effective in transforming B-lymphocytes and establishing latency. EBV remains hidden in a latent state until it senses triggers to activate the lytic phase. One such trigger is danger or threat to the host cell that is detected by the inflammasome, resulting in depletion of TRIM28, loss of heterochromatin on SZF1-bound viral DNA, and derepression of the viral latent-to-lytic switch protein [9]. While the existence of chromosomal SZF1 footprints at sites corresponding to the validated binding sites on the EBV genome suggest an additional level of regulation of EBV’s life cycle, our experiments do not support this idea. Instead, it seems that there is a clear dichotomy in the way SZF1 recognizes self versus foreign: it uses DNA sequence motif-based recognition of self versus distinct non-consensus sequences to target foreign DNA. Furthermore, these distinct sequences that silence viral lytic genes, appear not to be utilized by SZF1 to simultaneously silence host genes during latency.

The DNA binding specificity of SZF1 was previously investigated using an oligodeoxynucleotide library and recombinant SZF1 which identified the consensus sequence CCAGGGTAACAGCCG [18]. This sequence was not identified in our study or in another SZF1 ChIP-seq study in stem cells [2]. In this latter study, SZF1 was shown to control differentiation of stem cells by regulating the expression of differentiation genes. Based on informatic analysis of an SZF1 ChIP-seq dataset, its consensus binding site was predicted to be AATGGAAT though there was no functional validation of this site. In our experiments, none of the motifs bearing this consensus even when present in multiple copies was able to silence GFP; however, consensus sequences derived from two motifs that contained a variant, AATCGAAT, silenced GFP. While it is possible that AATGGAAT may not truly bind SZF1, it is more likely that both sequences are recognized by SZF1 but in different contexts. For instance, AATGGAAT may be used in stem cells but AATCGAAT used in terminally differentiated cells. Furthermore, while a KRAB-ZFP like SZF1 with four C2H2 zinc fingers is predicted to recognize 12nt binding sites, not every zinc finger needs to be engaged [17], thereby relaxing the sequence specificity. Likewise, nucleotide modification may be a factor. It is also noteworthy that the percent indexed reads for AATCGAAT remained constant at 0.1% between refractory and lytic cells (S6 Table), indicating that such constitutive heterochromatin on the cellular genome remains unchanged despite a major transition in the virus’s life cycle.

Only 2 out of 27 motifs were validated by the GFP assay. These motifs were derived from many peak pair midpoint sequences. As such, our motifs do not exactly match the sequences from which they were derived and likely explain the negative GFP assays. The GFP assay also does not consider the cell type, differentiation state, local proteome, and chromatin context of the original cells from which the motifs were derived. These are limitations of the GFP assay whose purpose was to only serve as a screening tool. Therefore, consensus sequences that failed to be validated by this assay may well be used by SZF1 to target the host genome.

Enrichment of SZF1 at the latent origin of replication on lytic genomes was unexpected. While the implications of this finding are unclear, we speculate that SZF1 may function at this site in at least two ways. First, its presence at oriP may physically exclude binding of telomere repeat factor 2 and origin recognition complex 1 [30], thereby preventing initiation of DNA replication from oriP in lytic cells. This would ensure replication of the viral genome strictly from the two oriLyts. Second, the presence of SZF1 at oriP may impair EBNA 1 binding to oriP and thereby, prevent EBNA1-mediated tethering of newly replicated genomes to chromosomes and in this way, ensure that replicated genomes are available for packaging. At a minimum, our finding of SZF1 at oriP in lytic cells hints at non-transcription related SZF1 functions exploited by a persistent virus.

Our findings point towards a mechanism by which a specific KRAB-ZFP, by targeting a consensus, may be responsible for constitutive heterochromatin on the centromeres of chromosomes 16 and 5. However, what lends specificity to SZF1 binding via non-consensus sequences on the viral genome is unclear. It is possible that the presence of other cell or viral proteins locally or recruited via changes in chromatin conformation provide specificity; this is supported by the larger than expected footprints of SZF1 surrounding its binding sites on *BZLF1p*, *BGLF4*, and *BDLF2* on the viral genome. This possibility might also extend to footprints identified from the host genome, as most motifs were unable to silence GFP. Perhaps SZF1-mediated silencing at the non-validated motifs is context dependent including the cell type, presence of other proteins, and chromatin setting. Indeed, with KRAB-ZFPs able to regulate heterochromatin and DNA methylation from several tens of kilobases away [8,14], binding of ZFPs to target DNA may be similarly modulated by proteins bound at distant sites.

The widespread presence of SZF1 footprints across the cellular genome was somewhat surprising particularly since only a small fraction of those footprints exhibited the binding motif. We had expected fewer footprints also because in contrast to ChIP-seq, which provides sequences of DNA in the vicinity of a bound protein, ChIP-exo yields sequences of protected DNA bound to protein, thus limiting the number of DNA peaks. One explanation for this high representation of footprints is that SZF1 may be recruited to the genome by other DNA-binding proteins that have their own binding sites. This may also explain why we were unable to validate most of the motifs using an extrachromosomal reporter in a non-B cell background.

In summary, the KRAB-ZFP SZF1 contributes to constitutive heterochromatin on the cellular genome while simultaneously silencing extrachromosomal foreign genomes. By using a motif to target self-DNA versus non-consensus-bearing sequences to target foreign genomes, it ensures integrity of the host genome even as it modulates the invader’s epigenome and regulates its life cycle.

## Materials and methods

### Study subjects and ethics statement

Peripheral blood mononuclear cells (PBMC) were isolated from the blood of healthy subjects at the University of Florida. Healthy subjects included two males and one female, with ages ranging from 19 to 27 years of age. Blood was drawn after obtaining written informed consent. The study of human subjects was approved by the Institutional Review Board at the University of Florida.

### Infection of PBMC

PBMC from healthy subjects were infected with EBV (wild type p2089 virus or mutated for SZF1-binding sites) at MOI of 1 in the presence of 20nM FK506. Cells were incubated with FK506 (AG Scientific) for an hour at 37° C before infection. Infected cells were left in culture to establish LCL.

### Cells lines and chemical treatment

EBV^+^ BL cell line HH514-16 (a gift from Dr. George Miller, Yale University) was maintained in RPMI 1640 supplemented with 10% fetal bovine serum (Gibco) and 1% penicillin-streptomycin (Gibco). LCL were generated and maintained as described before [31]. HEK-293T cell line (a gift from Dr. Erich Mackow, Stony Brook University) was maintained in DMEM supplemented with 10% fetal bovine serum and 1% penicillin-streptomycin. Sodium butyrate (NaB; 3mM; 303410, Sigma-Aldrich) was used to induce viral lytic cycle in HH514-16 cells.

### Plasmids, siRNAs, and transfection

BACmid p2089 was a gift from Professor Henri-Jacques Delecluse [26]. pEGFP-N1 was a gift from Dr. Nancy Reich at Stony Brook University.

All siRNAs targeting human transcripts were reconstituted with nuclease free water at a concentration of 10μM. Experiments performed using siRNA targeting *SZF1* were performed using two siRNAs (Table 1), with representative data shown.

**Table 1:** siRNAs targeting SZF1.

siRNA	Supplier	Catalog Number
SZF1-1	Dharmacon	J-020953-05-0005
SZF1-2	Dharmacon	J-020953-06-0005
Control siRNA (non-targeting)	Dharmacon	D001810-01-20

For nucleofection, cells were subcultured at 5x10^5^ cells/ml 24 h prior to transfection, washed twice with phosphate-buffered saline (PBS), and then 1x10^6^ cells were transfected with 20μg of plasmid or 200pmol siRNA in 100μl total Ingenio solution (MIR50117, Mirus) using an Amaxa Nucleofector II (program A-024). Cells were then seeded into pre-warmed complete medium at a concentration of 5x10^5^ cells/ml and harvested or further processed as indicated.

### EBV mutagenesis

EBV mutagenesis was performed using BAC recombineering as previously described, using primers listed in Table 2 [27].

**Table 2:** Primers used to generate mutations in the EBV BACmid p2089.

***BZLF1*p FP**	TATGAGGTACATTAGCAATGCCTGTGGCTCATGCATAGTTTCCAAGAGGGGCGGTGGCAGTTTTCAGAAGTGTCTAAAGGATGACGACGATAAGTAGGG
***BZLF1*p RP**	TTTTTGACACCAGCTTATTTTAGACACTTCTGAAAACTGCCACCGCCCCTCTTGGAAACTATGCATGAGCCACAGGAACCAATTAACCAATTCTGATTAG
***BZLF1*p Revert FP**	TATGAGGTACATTAGCAATGCCTGTGGCTCATGCATAGTTTCTAAAAGAGGAGGAGGCAGTTTTCAGAAGTGTCTAAAGGATGACGACGATAAGTAGGG
***BZLF1*p Revert RP**	ATTTTTGACACCAGCTTATTTTAGACACTTCTGAAAACTGCCTCCTCCTCTTTTAGAAACTATGCATGAGCCACAGGAACCAATTAACCAATTCTGATTAG
***BGLF4* (CDS) FP**	AGAGGCGATAGAGCTGCCGGCCCTTAGAAGACTTTAGCCGCAAGTCCATCATCTCGTTGCGGTCGTGGAGGGAAGCAGGATGACGACGATAAGTAGGG
***BGLF4* (CDS) RP**	TCCTGACTGATTATGGGACTGCTTCCCTCCACGACCGCAACGAGATGATGGACTTGCGGCTAAAGTCTTCTAAGGGAACCAATTAACCAATTCTGATTAG
***BGLF4* (CDS) Revert FP**	AGAGGCGATAGAGCTGCCGGCCCTTAGAAGACTTTAGCCGCACATCCAGCATCTTGTTGCGGTCGTGGAGGGAAGCAGGATGACGACGATAAGTAGGG
***BGLF4* (CDS) Revert RP**	TCCTGACTGATTATGGGACTGCTTCCCTCCACGACCGCAACAAGATGCTGGATGTGCGGCTAAAGTCTTCTAAGGGAACCAATTAACCAATTCTGATTAG
***BDLF2* (CDS) FP**	CAGGACCCTCTGCATATCTTGTACAAGGCGCCTTTCAACTCGTCCGCTTGCCCTGGTGACGTTAAATGTCCTGAGGATGACGACGATAAGTAGGG
***BDLF2* (CDS) RP**	CAGGCTGTGACTAATAGGAACAGGACATTTAACGTCACCAGGGCAAGCGGACGAGTTGAAAGGCGCCTTGTACAACCAATTAACCAATTCTGATTAG
***BDLF2* (CDS) Revert FP**	CAGGACCCTCTGCATATCTTGTACAAGGCGCCTTTCAACTCGGCCACTGGCTCTGGTGACGTTAAATGTCCTGAGGATGACGACGATAAGTAGGG
***BDLF2* (CDS) Revert RP**	CAGGCTGTGACTAATAGGAACAGGACATTTAACGTCACCAGAGCCAGTGGCCGAGTTGAAAGGCGCCTTGTACAACCAATTAACCAATTCTGATTAG
***BcLF1* (CDS) FP**	GGTGCATGGTGGCAGCCACTCGCGGGTCCCCGTAAAACATGTGAAATGGTATTGCGTGAAAGAGACACTGGGTGAGGATGACGACGATAAGTAGGG
***BcLF1* (CDS) RP**	TCCGAGAGGACCCGGGCCGTCACCCAGTGTCTCTTTCACGCAATACCATTTCACATGTTTTACGGGGACCCGCGAACCAATTAACCAATTCTGATTAG

### Generation of BAC-EBV

EBV preps were generated from 293-BAC cells harboring wild-type p2089 or mutant genomes via transfection of overexpression plasmids containing *BZLF1* and *BRLF1* open reading frames. After 5 days, supernatants were collected and filtered for further use.

### Antibodies

Antibodies include goat anti rabbit SZF1/ZNF589 (S-14, sc-100263, Santa Cruz; for ChIP-exo procedure), goat anti-rabbit SZF1/ZNF589 (PA5-68941, ThermoFisher), normal rabbit IgG (sc-2027, Santa Cruz), mouse anti-EA-D Ab (MAB8186, EMD), HRP conjugated goat anti-mouse IgG (H+L) (AP308P, EMD Millipore), and HRP conjugated goat anti-rabbit IgG (H+L) (AP307P, EMD Millipore). All antibodies were used at concentrations and conditions recommended by manufacturers.

### ChIP-exo

1x10^8^ HH514-16 cells per replicate were treated with NaB or left untreated. After 24 hours, cells were washed twice with 1x PBS before performing Fluorescence Activated Cell Sorting (FACS) using human sera as previously reported [21]. Lytic and refractory gates were placed on NaB-treated cells based on staining with reference EBV-seropositive serum after comparing to similarly-treated cells stained with reference EBV-seronegative serum. Sorted lytic and refractory cells were recovered, washed twice with 1x PBS, and pelleted cells from 3 independent experiments sent for ChIP-exo processing at Peconic Genomics [24]. Briefly, sequencing reads generated from the Chip-exo analysis were trimmed and filtered to remove barcodes and low quality reads. The clean reads from untreated, refractory and lytic samples were mapped to human (Hg38) and EBV (NC_007605) reference genome, respectively, with Bowtie 2 [32]. The resulting SAM files were sorted, indexed and then converted to BAM files with SAMtools 1.9 [33]. Peaks were called using the GeneTrack [34] and GEM [35] algorithms using the default parameters of Fine and Coarse grain peak-calling (sigma and exclusion zone): s = 5, e = 10 and s = 20, e = 40, respectively, of the GeneTrack algorithm. The binomial distribution of reads that fit to a Gaussian distribution were used to identify peak summits and the number of reads associated with each peak were extracted. Singleton peaks with read standard deviation of zero (read SD = 0) were removed to retain high confidence reads that were used for peak pairing of biological replicates. To improve motif discovery, peaks were also called using the GEM algorithm based on the binding event locations and predictions using k_seq = 5000, k_win = 61, q = 3, smooth = 3. (k_seqs: the number of top-ranking events, k_win: the sequence window size around the binding event, q: significance level for q-value, specified as log10 (q-value), smooth: the width (bp) to smooth the read distribution). Sequences underneath the detected peaks within ±30 bp of the midpoint of the paired peaks were extracted and analyzed using MEME-ChIP [5] tools on the MEME-suite 5.0.5 [6] algorithm for motif discovery. Midpoint sequences contributing to motif 1 were plotted on the human genome in R using Circos [36]. For initial prediction of EBV genome binding sites, 15nt on either side of the midpoint coordinate of each peak-pair was considered, yielding predicted binding sites of 31nt each. Read distributions at SZF1-binding sites and oriP shown in Figs 8 and S1 were plotted in R using read coverage values from sorted lytic and refractory genomes determined with BEDTools software (v2.30.0) [37].

### ChIP-PCR

DNA from 4x10^6^ cells per replicate was crosslinked by adding 37% formaldehyde to cells for 10 min at a final concentration of 1% followed by the SimpleChIP Enzymatic Chromatin IP Kit protocol (9003, Cell Signaling). One to 2% of DNA was set aside as input. Antibodies used for pulldown were goat anti rabbit SZF1/ZNF589 (S-14, sc-100263, Santa Cruz), and normal rabbit IgG (sc-2027, Santa Cruz). All antibodies were used at 5 μg. Isolated chromatin was used for quantitative PCR analysis of oriP and *BZLF1* DNA. The following primer sets were used: oriP- AGATATTTGGGTAGTATATGCTAC FP, GCTATCCTAATCTGTATCCGGGT RP; *BZLF1*p- TTCAGCAAAGATAGCAAAGGT FP, ACTTCTGAAAACTGCCTCCT RP. Data were normalized to input and normal rabbit IgG controls for analysis.

### Isolation of proteins on nascent DNA (iPOND)

1×10^8^ HH514-16 cells were induced with 3mM NaB with or without phosphonoacetic acid (PAA) (200 μg/ml) for 36 hours prior to performing iPOND as described previously [29]. Briefly, cells were pulsed with 10 μM EdU for 15 min, spun down, cross-linked with 1% formaldehyde for 20 min, and quenched with 0.125 M glycine for 5 min. For click chemistry, cells were incubated with 10 μM biotin-azide in click reaction buffer for 2 hours. Nuclei were isolated and digested with 1 μL of micrococcal nuclease (10011, Cell Signaling) at 37°C for 20 min and suspended in cold lysis buffer (1% SDS, 50 mM Tris, pH 8.0), and subjected to sonication using a microtip sonicator to break nuclear membranes. After removing debris, the supernatant was incubated with 100 μL of streptavidin agarose beads (69203, EMD Millipore) overnight at 4°C and washed three times with lysis buffer and one time with 1M NaCl. Protein-DNA complexes were eluted with 2X Laemmli buffer at 95°C for 25 min and subjected to immunoblotting.

### Immunoblotting

Cells were lysed for immunoblotting using RIPA buffer [50mM Tris-HCl (pH 7.4), 150mM NaCl, 1% (v/v) NP40, 1% (w/v) deoxycholate, 1mM EDTA, 1X protease and phosphatase inhibitor cocktail (catalog no. 5872, Cell Signaling Technology)]

Cell extracts were electrophoresed in 10% SDS-polyacrylamide gels, transferred onto nitrocellulose membranes, and blocked using 5% milk. Immunoblotting was performed using indicated antibodies at concentrations recommended by the manufacturer.

### Flow cytometry

HEK-293T cells were washed twice with 1x PBS and then subjected to flow cytometry. Flow cytometry data were acquired on a ThermoFisher Attune NxT Flow and analyzed using FlowJo software (Tree Star) with placement of GFP^+^ and GFP^-^ gates based on parallel untransfected control samples. As a transfection control in these experiments, cells were co-transfected with SignalSilence Control siRNA Cy5 Conjugate (86921, Cell Signaling). FACS of HH514-16 cells for ChIP-exo was performed using a BD Aria II FACS instrument; again, staining and gate placement were performed as described in ChIP-Exo.

### Quantitative reverse transcriptase-PCR (RT-qPCR)

Total RNA was isolated from HH514-16 cells by using an RNeasy kit (Qiagen) followed by DNase digestion (Promega). RNA was quantitated by using a NanoDrop instrument (Thermo Scientific). RNA (1μg) was converted to cDNA by using MuLV Reverse Transcriptase (New England Biolabs). Relative transcript levels of selected cellular genes were determined with gene-specific primers (listed in Table 3) by using Fast SYBR green Master Mix on a Quant Studio 3 thermocycler (Applied Biosystems) and analyzed using the ΔΔCT method.

**Table 3:** Sequences of primers used for RT-qPCR.

***18S rRNA*** FP: GTAACCCGTTGAACCCCATT	RP: CCATCCAATCGGTAGTAGCG
***BZLF1*** FP: TTCCACAGCCTGCACCAGTG	RP: GGCAGAAGCCACCTCACGGT
***BGLF4*** FP: CGGTTTGAGCACCCTCATCT	RP: GGCAAACGTGTAGGAGGTCA
***BDLF2*** FP: GTCCCAACAACTTCCAACGC	RP: ATTGCTAGTCACACCCGTGG
***BcLF1*** FP: CCTCTTGGAATGCAGCTGGGGCCAG	RP: TTTTACCAGGGACGAGGACA
***ANKRD26P1*** FP: AGGTGGAGTGCATCCTTTCG	RP: CTACCACTTCTGGATGGCCG
***SZF1*** FP: TCCAAATCCTCCTAACCCCT	RP: GAGCAGCTACTGGGCTGG
***LOC107986671*** FP: GCGCATCACTCCGATCTGTA	RP: GGACAGTCACAGATGGTCGG
***LOC107984659*** FP: CCCAGGAGAAGCAGTGTGTT	RP: TCACAGCCTCAACCCAACAG
***TIGD4*** FP: GAGAGCACGAAGAAGAAGAAGA	RP: AAGTTTTCATGCAACTATTACCCA
***BALF1*** FP: TGCCACGCCCATTTTATC	RP: GGTCATCCAGGTAGTTTCGC
***RAMACL*** FP: TTCTTTTTCTTTCCTCCTCTGGT	RP: CGTGGAACTCCAGACCACTC
***NSA2P6*** FP: CGCAACATACACCATAAAAGGC	RP: GCCCTTGGGGTGCAAAAATG
***ARFIP1*** FP: AGAGCGGCGGAAAGGATAAG	RP: TGAGCCATGGTAGACTCCTTTC
***DYNC1H1*** FP: CAGGACATAGACCTGTCGCC	RP: TGGTGGGAACTCGACAGTTG
***PPP2R5C*** FP: GAGGCTCATCAGGCACAGAA	RP: AAGGCTTTCTTGGTGTGGGG
***MIR1913*** FP: TCTACCTCCCGGCAGAGG	RP: CCAGCCACTTGGCAGCA

### Assay for EBV load

Viral DNA in supernatant was measured using quantitative-PCR (qPCR) by amplifying EBV *BALF5* gene with forward primer–CGTCTCATTCCCAAGTGTTTC and reverse primer–GCCCTTTCCATCCTCGTC. Released EBV particles were assayed by treating supernatants of newly generated 293-BAC cells with 1μg/μl RQ1 RNase-free DNase (M610A, Promega) and then Proteinase K overnight at 37°C. Absolute EBV genome copy number was determined with a standard curve obtained through qPCR using serially diluted BACmid p2089 as template. Primers targeting the EBV *BALF5* gene were used to calculate relative released virus compared to wild type 293-BAC cells.

### Growth curve analysis

To assess changes in growth of primary B cells infected with wild type p2089 virus or SZF1-binding site mutant viruses, 5x10^6^ primary B cells from three individual donors were infected at an MOI of 1 (calculated via *BALF5* standard curve qPCR) with each respective virus. Live cell counts were performed beginning on day 14 post infection.

### Statistical analysis

*P* values were calculated by comparing the means of two groups of interest using unpaired Student t test.

## Supporting information

S1 Fig Read coverage at validated SZF1-binding sites on the EBV genome. Plots show read distributions at SZF1-binding sites and +/- 60 bp mapping to *BZLF1*p, BGLF4, and BDLF2. Coverage of the reads from lytic and refractory genomes were determined with Bedtools software (v2.30.0) and plotted in R. The lytic reads were normalized to refractory EBV genome copy number. Rightward and leftward strands are indicated by blue and red, respectively. Genome position numbers corresponding to the reference genome NC_007605 are indicated and validated SZF1 binding sites are indicated with brackets.
(TIF)

S1 Table SZF1 ChIP-exo reads mapped to human and EBV genomes. Total SZF1 ChIP-exo reads mapping to the human genome reference (Hg38/GRCh38) or EBV genome reference (NCBI Accession: NC_007605) for each experimental condition (lytic, refractory, and untreated samples). Mapped and unique lytic reads were normalized based on EBV copy number in refractory cells.
(XLSX)

S2 Table ChIP-exo peak calling. SZF1 ChIP-exo sequence reads were mapped to either the EBV (NC_007605) or human (GRCh38) genome. Total indexed reads and unique read counts are shown for ChIP-exo sequence reads contributing to peak calling. **Peaks:** overlapping clusters of reads with sigma = 5 exclusion zone = 10 (s5e10F1), or sigma = 20 exclusion zone = 40 (s20e40F1), excluding singletons. **Singletons:** peaks wherein all associated reads are located at a single chromosomal coordinate. **Peak-median:** median read count for all peaks, excluding singletons. **Peak-mean:** average read count for all peaks, excluding singletons. **Median-std:** median standard deviation for all peaks with standard deviation greater than zero. The standard deviation reflects the variance of read positions within each peak. A peak with a standard deviation = 0 defines a singleton peak and is excluded from further analysis. **Mean-std:** average standard deviation for all peaks with standard deviations greater than zero.
(XLSX)

S3 Table Putative SZF1-binding sites on the EBV genome in B cells refractory to lytic viral replication. Peak midpoint positions mapped to the EBV reference genome (NC_007605), listed in descending order based on number of contributing midpoint sequences. Immediate early, early, and late refer to kinetic classes of lytic genes; CDS, coding sequence. Sites corresponding to the bolded genes were analyzed via GFP repression assay in Fig 2.
(XLSX)

S4 Table Validated SZF1-binding sites with 12 contiguous nts predicted or footprinted on the human genome. SZF1 binding sites within the EBV genome were mapped to the human genome with the requirement of 12 contiguous nucleotides sharing 100% identity. Neighboring genes within 100 kb of the potential binding sites, with the potential to be regulated via SZF1 binding, are shown. Sites with validated presence in peak-pair sequences, i.e. footprints determined from the SZF1 ChIP-exo data for each condition (untreated [U], refractory [R], or lytic [L]) are also shown.
(XLSX)

S5 Table Consensus sequences derived from the top 27 motifs in the SZF1 ChIP-exo library. Sequences derived from the top 27 motifs identified from Meme-suite analysis of SZF1 ChIP-exo read libraries mapped to Human (GRCh38) or EBV (NC_007605) genomes. Note: Fine grain peak-calling when sigma (s) = 5, exclusion zone (e) = 10; Coarse grain peak-calling sigma (s) = 20, exclusion zone (e) = 40; Untreated Human s5e10 and Lytic EBV s5e10 resulted in no motif hits. e values in bold indicate validated Motifs 1 and 2 while e value highlighted in grey indicates Motif 3 depicted in Fig 6. Core 8 nt sequences of AATGGAAT and AATCGAAT are highlighted in yellow and green, respectively with the overlapping region highlighted in grey.
(XLSX)

S6 Table Human mapped reads matching to core 8 mers in motifs 1 and 2. (XLSX)

S7 Table List of SZF1 footprints, represented by 176 peak-pairs on the human genome, contributing to motif 1. (XLSX)

S8 Table Genes within 100 kb of motif 1-bearing footprints on chromosomes 16 and 5. Start and end positions of motif 1 consensus (ATGGAATCGAATGGAATC) clustered on chromosomes 16 and 5 of the human genome (GRCh38) are shown along with any genes found within 100 kb upstream or downstream of the motif clusters. Additional SZF1 motif 1 clusters are shown in Fig 7B.
(XLSX)
