# Supplementary figures and images for: A heterochromatin inducing protein differentially recognizes self versus foreign genomes
Source: PLoS Pathog. 2021 Mar 17;17(3):e1009447. doi: 10.1371/journal.ppat.1009447 (PMC8007004; doi:10.1371/journal.ppat.1009447)

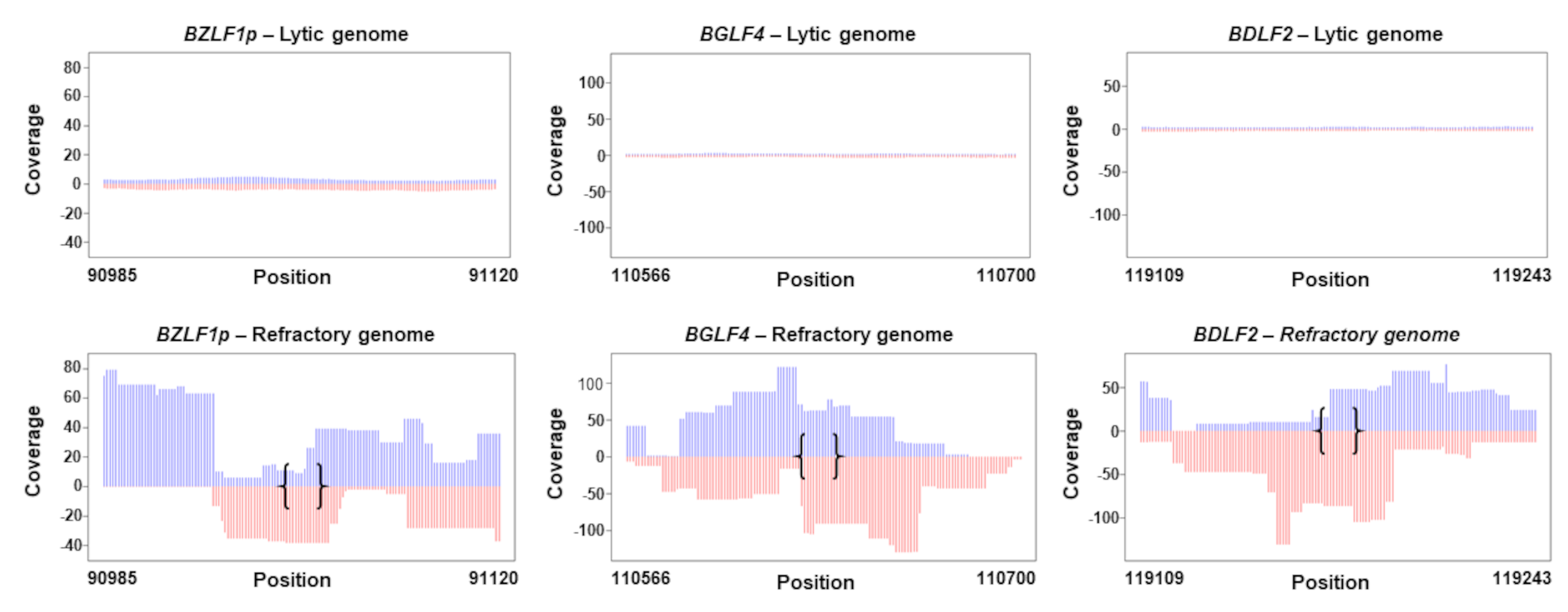

Supplement: S1 Fig — Plots show read distributions at SZF1-binding sites and +/- 60 bp mapping to BZLF1p, BGLF4, and BDLF2. Coverage of the reads from lytic and refractory genomes were determined with Bedtools software (v2.30.0) and plotted in R. The lytic reads were normalized to refractory EBV genome copy number. Rightward and leftward strands are indicated by blue and red, respectively. Genome position numbers corresponding to the reference genome NC_007605 are indicated and validated SZF1 binding sites are indicated with brackets. (TIF) [file ppat.1009447.s001.tif]
